# Overall reduced lymphocyte especially T and B subsets closely related to the poor prognosis and the disease severity in severe patients with COVID-19 and diabetes mellitus

**DOI:** 10.1186/s13098-020-00622-3

**Published:** 2021-01-12

**Authors:** Dafeng Liu, Yong Wang, Bennan Zhao, Lijuan Lan, Yaling Liu, Lei Bao, Hong Chen, Min Yang, Qingfeng Li, Yilan Zeng

**Affiliations:** 1 Department of Internal Medicine, the Public and Health Clinic Centre of Chengdu, No. 18 Jingjusi Road, Jinjiang District, Chengdu, 610066 Sichuan China; 2 Department of the Severe Second Ward, The Public and Health Clinic Centre of Chengdu, Chengdu, China; 3 Department of the Tuberculosis First Ward, The Public and Health Clinic Centre of Chengdu, Chengdu, China; 4 Department of Clinical Medical Laboratory, The Public and Health Clinic Centre of Chengdu, Chengdu, China; 5 Department of Vice President’s Office, The Public and Health Clinic Centre of Chengdu, No. 18 Jingjusi Road, Jinjiang District, Chengdu, 610066 Sichuan China

**Keywords:** Lymphocyte subsets, Coronavirus disease 2019 (COVID-19), Diabetes mellitus, Prognosis, Disease severity

## Abstract

**Background:**

A dysregulated host immune response is common in patients with COVID-19.

**Aim:**

In this study, we aimed to define the characteristics of lymphocyte subsets and their relationship with disease progression in COVID-19 patients with or without diabetes mellitus (DM).

**Methods:**

The baseline peripheral lymphocyte subsets were compared between 55 healthy controls and 95 patients with confirmed COVID-19, and between severe and non-severe COVID-19 patients with or without DM.

**Results:**

The prevalence of DM in the COVID-19 group was 20%, and patients with severe COVID-19 had a higher prevalence of DM than those with non-severe disease (*P* = 0.006). Moreover, a significantly poor prognosis and a higher rate of severity were found in those with DM relative to those without DM (*P* = 0.001, 0.003). Generally, all lymphocytes and subsets of lymphocytes, especially B and T cells, were significant reduced in COVID-19 patients, particularly in those with DM. Patients with severe COVID-19 and DM had the lowest lymphocyte counts compared with those with severe COVID-19 without DM, and those with non-severe COVID-19 with or without DM. Partially decreased lymphocyte subsets, age and DM were closely related to disease progression and prognosis.

**Conclusions:**

These findings provide a reference for clinicians that immunomodulatory treatment may improve disease progression and prognosis of COVID-19 patients, especially those with severe disease with DM.

*Trial registration* Chinese Clinical Trial Register ChiCTR2000034563

## Introduction

Despite the worldwide pandemic [1–6], most patients with coronavirus disease 2019 (COVID-19) have a good prognosis, but some patients with rapid disease progression have a poor prognosis [7–11]. The mortality rate in China is approximately 3.5 ~ 5.56%, [6, 12] and diabetes mellitus (DM) as a comorbidity could promote disease progression and worsen the prognosis [8, 9].

Dysregulation of the host immune response, especially reduced cellular immunity, plays an important role in the pathophysiology of COVID-19 [8, 13, 14], SARS [15], and MERS-CoV [16]. Severe acute respiratory syndrome coronavirus 2 (SARS-CoV-2) can alter both the innate and adaptive immune responses [17]. Reduced CD3+, CD4+, and CD8+ T lymphocyte counts may reflect the severity of COVID-19 [18].

DM could lead to dysfunctional cellular immunity [19], and DM was also shown to be an independent risk factor for the prognosis of COVID-19 [20].

The effects of concomitant COVID-19 and DM on all lymphocytes and lymphocyte subsets as well as, on the characteristics of those subsets and their relationship with disease progression and prognosis in COVID-19 patients with or without DM are unknown and worthy of investigation.

## Methods

### Subjects

This study had a cross-sectional research design.

**Fig. 1:**
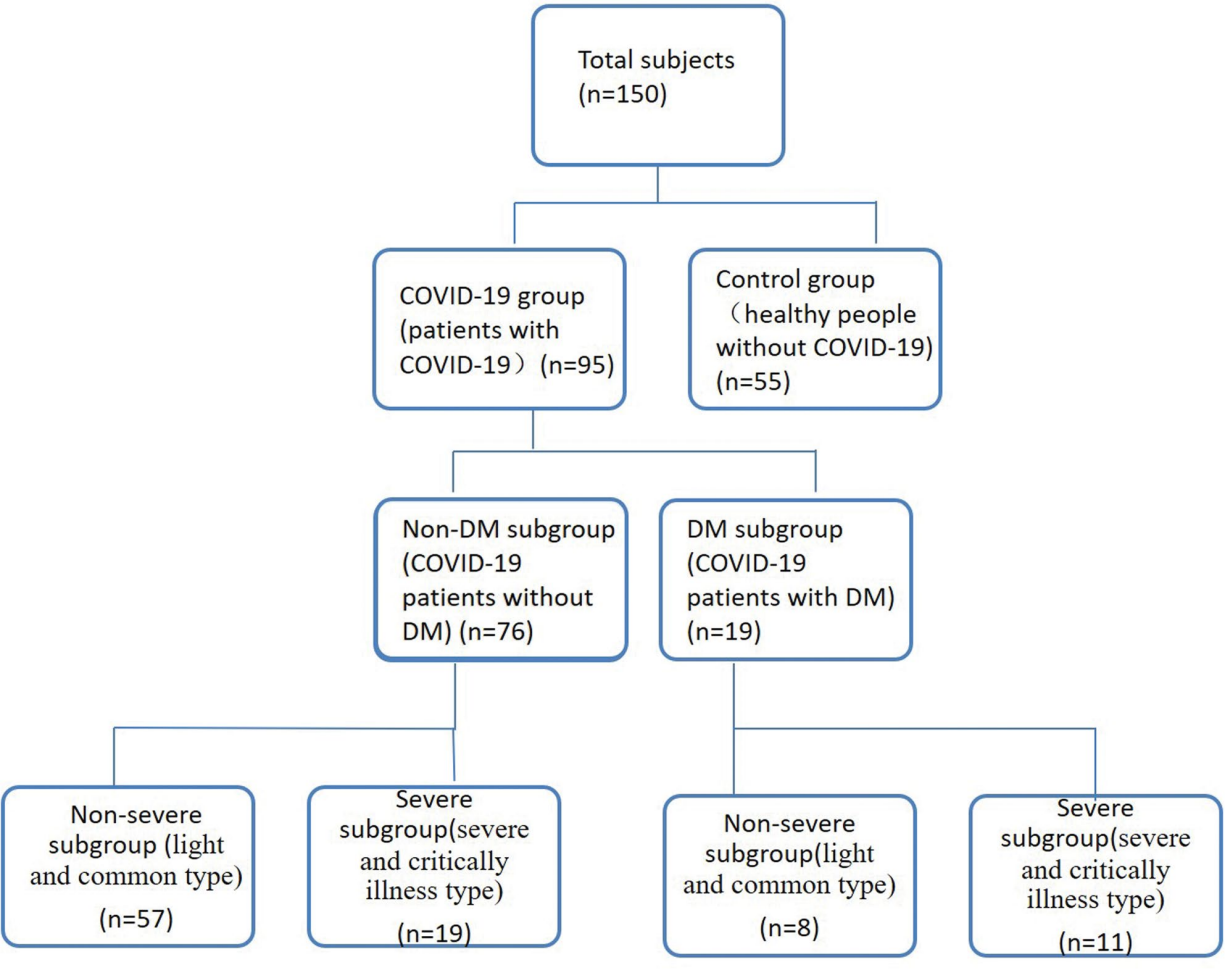
Patient data. Non-severe refers to the clinical type of COVID-19 that is most common. Severe refers to the clinical type of COVID-19 that is associated with severe and critical illness. DM, diabetes mellitus; non-DM, without diabetes mellitus

In all, 95 COVID-19 patients, from the hospital isolation ward [14], and 55 healthy controls, from the medical examination clinic, who presented the Public and Health Clinic Centre of Chengdu from January 16, 2020, to March 16, 2020, were retrospectively recruited (Fig. 1). The Ethics Committee of the Public and Health Clinic Centre of Chengdu approved this study (ethic approval number: PJ-K2020-26-01). Written informed consent was waived by the Ethics Commission of the designated hospital because this study is related to emerging infectious diseases.

### Clinical typing, disease diagnosis and cured criteria

The criteria of COVID-19 clinical typing, disease diagnosis and cure were based on the seventh Trial Version of the Novel Coronavirus Pneumonia Diagnosis and Treatment Guidance [7].

The DM diagnostic criteria were adopted from the Guidelines for the Prevention and Treatment of Type 2 Diabetes in China (2017 edition) [21].

### Grouping standards

Ninety-five COVID-19 patients were enrolled in the COVID-19 group and 55 healthy controls were enrolled in the control group.

Of the 95 COVID-19 patients 76 and 19 cases were divided into the non-DM subgroup (patients without DM), and the DM subgroup (patients with DM), respectively. Of these patients 57, 19, 8 and 11 cases were further divided into the non-severe non-DM subgroup (those with non-severe COVID-19 without DM), the severe non-DM subgroup (those with severe COVID-19 without DM), the non-severe DM subgroup (those with non-severe COVID-19 and DM), the severe DM subgroup (those with severe COVID-19 and DM), respectively (Fig. 1).

### The definition of the viral negative conversion time and the prognosis [7]

The three prognosis were death, unhealed and cured.

The discharge standard was normal body temperature over three days, obvious improvement of respiratory symptoms, obvious improvement in lung imaging, and a negative nucleic acid test in two consecutive respiratory specimens over at least a 24-h interval.

The viral negative conversion time was defined as the time from onset to the first negative nucleic acid test that met the discharge criteria.

### Data collection

Demographic data, clinical data, lymphocyte counts and subset counts of all 150 cases were collected and used, to establish databases. Researchers strictly controlled the accuracy, authenticity and completeness of the data.

### Statistical analysis

Statistical analyses were performed using GraphPad Prism 8 (GraphPad, CA, the USA). The measurement data are expression as x ± SD, and ANOVA was used for multi-group comparison of the homogeneity of variance and normally distributed data. A least significant difference (LSD) *t*-test was used for further comparisons between two groups. When the data did not have the homogeneity of variance and normal distribution, an independent sample Kruskal–Wallis H(K) test was used for multi-group comparisons, while a Mann–Whitney U test was used for further comparisons between two groups. An independent-sample *t*-test were used to make comparisons between two groups. A percentage or proportion was used to express enumeration data, and a Chi-square test was used for comparisons of these data. Spearman correlation analysis was used for the two-factor correlation analysis, and multiple stepwise regression was used for the multi-factor correlation analysis. Statistical significance was defined as *P* < 0.05.

## Results

### Baseline conditions

Patients in the COVID-19 group were significantly younger than those in the control group, and patients in the non-DM subgroup were younger than those in the DM subgroup, these differences were statistically significant (Table 1). However no significant difference in age were found between the control group and the COVID-19 DM subgroup (Table 1), and no significant difference in gender were found between the control group and the COVID-19 group or, between the COVID-19 non-DM subgroup and the COVID-19 DM subgroup (Table 1) (all *P* > 0.05).

**Table 1:** Comparison of the baseline conditions among the three groups(*n* = 150)

Variable	Control group (*n* = 55)	COVID group (*n* = 95)			*χ*^*2*^ or *F* score	*P* score
		Total (*n* = 95)	Non-DM subgroup (*n* = 76)	DM subgroup (*n* = 19)		
Age (year)	55.54 ± 7.79	49.13 ± 17.13**	46.75 ± 17.16***	60.22 ± 11.88^##^	*F* = 6.272	0.0004
Male (case, %)	25 (45.45)	46 (48.42)	36 (47.37)	10 (52.63)	*χ*^*2*^ = 0.290	0.865

DM, diabetes mellitus; non-DM, without diabetes mellitus. Unpaired one-way ANOVA was used for intergroup comparisons of age. Chi-square test was used for comparisons of gender. Unpaired *t*-test was used for comparisons of age between the control group and the COVID-19 group, COVID-19 non-DM subgroup and COVID-19 DM subgroup, **P < 0.001, ***P < 0.001. Unpaired *t*-test was used for comparisons of age between the latter two subgroups, ^##^P < 0.01

**Fig. 2:**
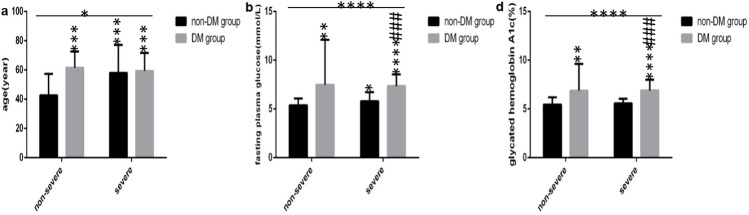
Comparison of age, FPG and HbA1c among the four subgroups. DM, diabetes mellitus; non-DM, without diabetes mellitus; COVID-19, coronavirus disease 2019; FPG, fasting plasma glucose; HbA1c, glycated hemoglobin A1c. **a** age. **b** FPG levels. **c** HbA1c levels. Unpaired two-way ANOVA was used for interaction comparisons (**a** *P* < 0.05; **b**, **c**, *P* all > 0.05). Unpaired one-way ANOVA was used for intergroup comparisons (**a** *P* < 0.05; **b**, **c** *P* all < 0.0001). Unpaired *t*-tests was used for comparison with the non-severe non-DM subgroup, *P < 0.05, **P < 0.01, ***P < 0.001, ****P < 0.0001. Unpaired *t*-tests was used for comparison with the severe non-DM subgroup, ^###^*P* < 0.001

The COVID-19 patients in the non-severe non-DM subgroup were significantly younger than those in the other three subgroups, and patients in the other three subgroups were similar in age (Fig. 2a). No significant difference in gender were found among the four subgroups (Table 2).

**Table 2:** Comparison of the baseline conditions among the four COVID-19 subgroups(*n* = 95)

Variable	Non-DM group (*n* = 76)		DM group (*n* = 19)		*χ*^*2*^or *F* score	*P* score
	non-severe subgroup (*n* = 57)	severe subgroup (*n* = 19)	non-severe subgroup (*n* = 8)	severe subgroup (*n* = 11)		
Male (case, %)	25 (43.86)	11 (57.89)	3 (37.50)	7 (63.64)	*χ*^*2*^ = 2.532	0.469

Chi-square test was used for gender comparisons

DM, diabetes mellitus; non-DM, without diabetes mellitus

Compared with patients in the DM subgroup, patients in the non-DM subgroup had lower fasting plasma glucose (FPG) level, which reflects the fasting blood glucose level on that day. Those patients also had lower hemoglobin A1c (HbA1c) level, which reflects the overall blood glucose levels in the last three months (Fig. 2b, c). Moreover patients in the non-severe non-DM subgroup had the lowest FPG levels (Fig. 2b), and patients in the severe non-DM subgroup had lower FPG levels and HbA1c levels (Fig. 2b, c) than those in the severe DM subgroup. However the FPG and HbA1c levels (Fig. 2b, c) were similar between patients in the non-severe DM subgroup and those in the severe DM subgroup. The HbA1c levels (Fig. 2c) were also similar between patients in the non-severe non-DM subgroup and those in the severe non-DM subgroup.

### The prevalence of DM and the rate of severity in COVID-19 patients

The prevalence of DM in the COVID-19 group was 20% (19/95), and those patients with severe disease had a higher the prevalence of DM than those with non-severe disease (*P* = 0.006). Moreover the rate of severity in the non-DM subgroup was lower than that in the DM subgroup (*P* = 0.003), and all differences were significant.

### The characteristics of baseline lymphocytes and subsets in COVID-19 patients

**Fig. 3:**
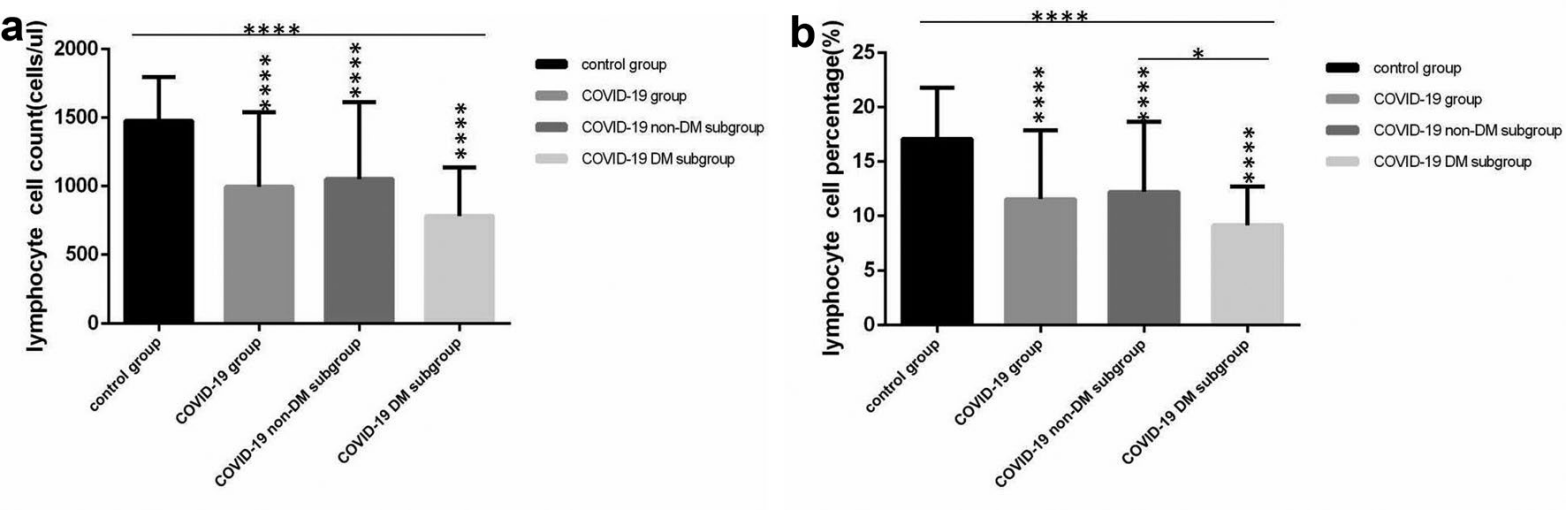
Comparison of lymphocyte counts and percentages among the four subgroups. DM, diabetes mellitus; non-DM, without diabetes mellitus; COVID-19, coronavirus disease 2019. **a** Lymphocyte count. **b** lymphocyte percentage. Unpaired one-way ANOVA was used for intergroup comparisons (**a**, **b**, *P* all < 0.0001). Unpaired *t*-tests was used for comparisons with the control group or between two groups, *P < 0.05, ****P < 0.0001

**Fig. 4:**
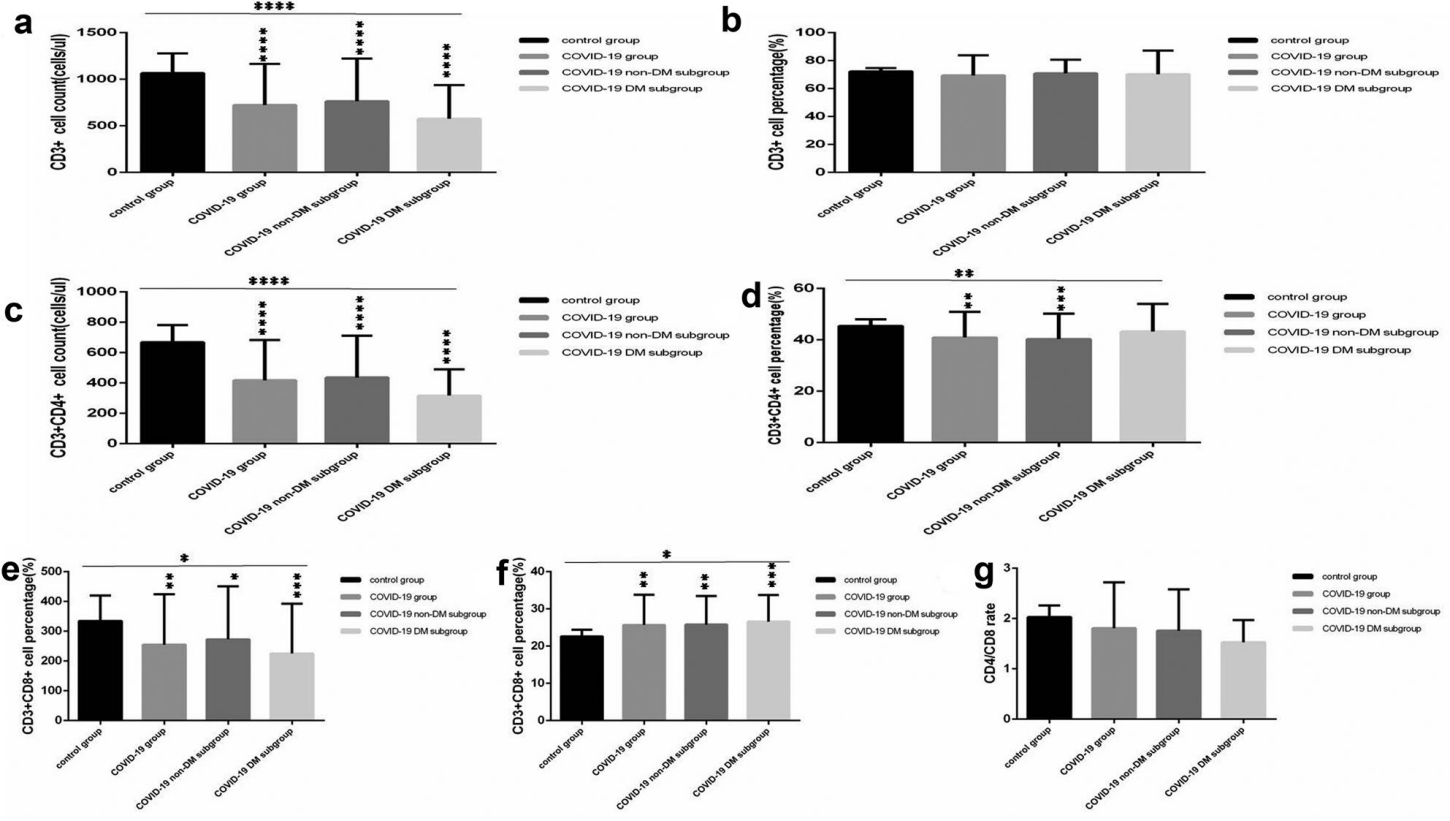
Comparison of T lymphocyte subset counts and percentages among the four subgroups. DM, diabetes mellitus; non-DM, without diabetes mellitus; COVID-19, coronavirus disease 2019. **a** CD3+ Cell count. **b** CD3+ Cell percentage. **c** CD3+ CD4+ cell count. **d** CD3+ CD4+ cell percentage. **e** CD3+ CD8 + cell count. **f** CD3+ CD8 + cell percentage. G. Ratio of CD4 + /CD8 + cells. Unpaired one-way ANOVA was used for intergroup comparisons (**a**, **c**, *P* all < 0.0001; **d** *P* < 0.01; **e**, **f** *P* all < 0.05). Unpaired *t*-tests was used for comparisons with the control group, *P < 0.05, **P < 0.01, ***P < 0.001, ****P < 0.0001

**Fig. 5:**
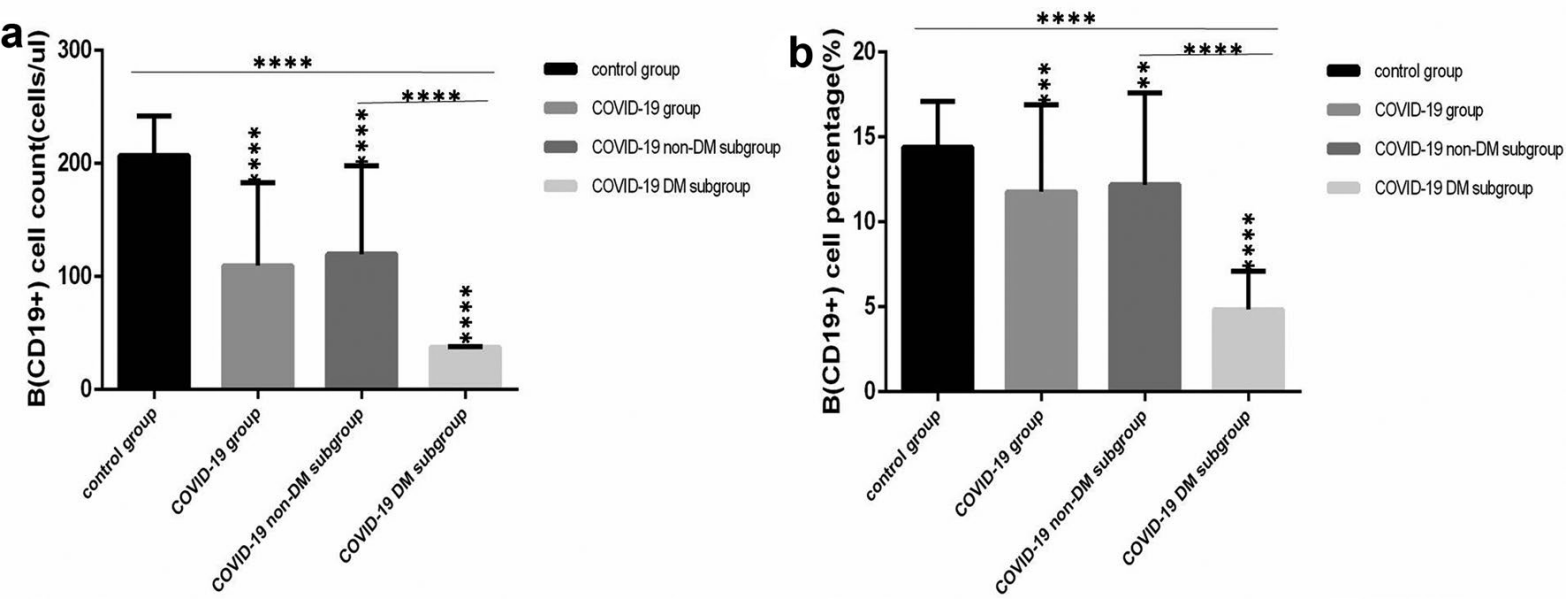
Comparison of B lymphocyte count and percentage among the four subgroups. DM, diabetes mellitus; non-DM, without diabetes mellitus; COVID-19, coronavirus disease 2019. **a** B lymphocyte count. **b** B lymphocyte percentage. Unpaired one-way ANOVA was used for intergroup comparisons (**a**, **b** *P* all < 0.0001). Unpaired *t*-tests was used for comparisons with the control group or between two subgroups, **P < 0.01, ***P < 0.001, ****P < 0.0001

**Fig. 6:**
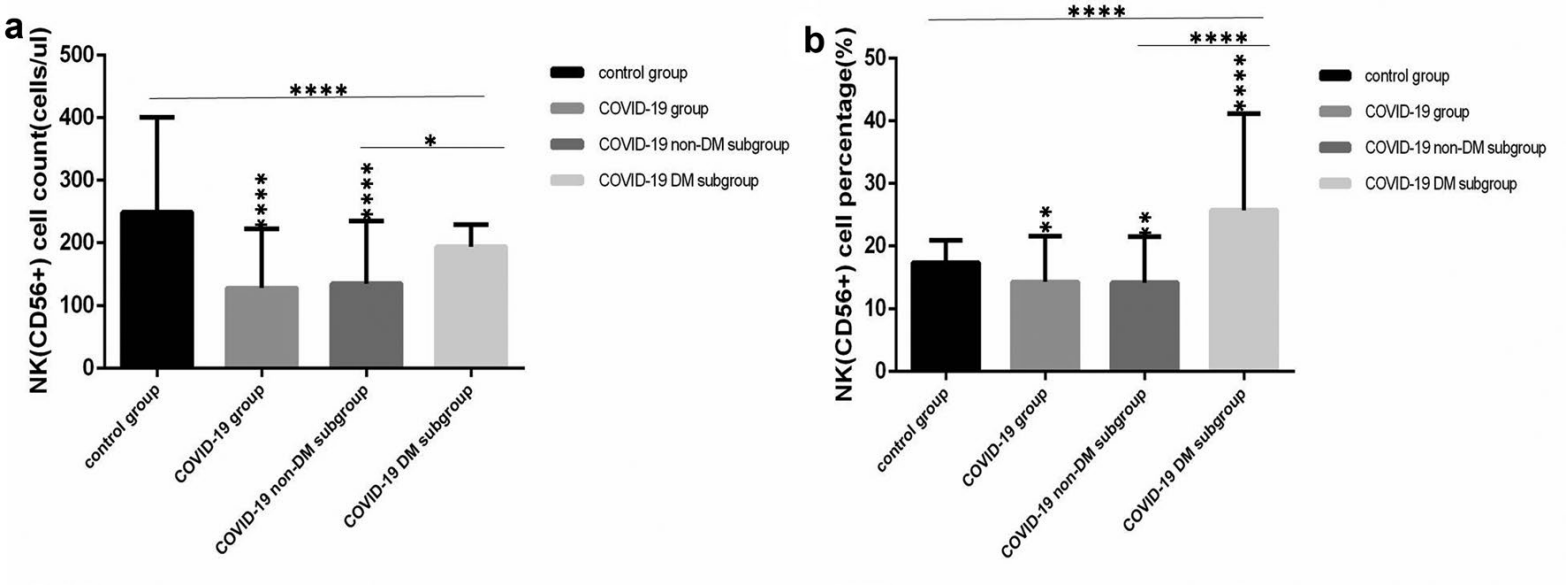
Comparison of NK lymphocyte count and percentage among the four subgroups. DM, diabetes mellitus; non-DM, without diabetes mellitus; COVID-19, coronavirus disease 2019. **a** NK lymphocyte count. **b** NK lymphocytes percentage. Unpaired one-way ANOVA was used for intergroup comparisons (**a**, **b** *P* all < 0.0001). Unpaired *t*-tests was used for comparisons with the control group or between two subgroups, *P < 0.05, **P < 0.01, ****P < 0.0001

In the COVID-19 group at baseline, the lymphocyte counts and percentages (Fig. 3a, b), CD3+ cell counts (Fig. 4a), CD3+ CD4+ cell counts and percentages (Fig. 4c, d), CD3+ CD8+ cell counts (Fig. 4e), B(CD19+) cell counts and percentages (Fig. 5a, b), and NK (CD56+) cell counts and percentages (Fig. 6a, b) were all lower than the corresponding values in the control group. Moreover, in the COVID-19 DM subgroup, the lymphocyte counts and percentages (Fig. 3a, b), CD3+ cell counts (Fig. 4a), CD3+ CD4+ cell counts (Fig. 4c), CD3+ CD8 + cell counts (Fig. 4e), B(CD19+) cell counts and percentages (Fig. 5a, b) were lower, but NK (CD56+) cell counts and percentages (Fig. 6a, b) were higher than the corresponding values in the COVID-19 non-DM subgroup. These findings were, especially evident in the lymphocyte percentages, B(CD19+) cell counts and percentages, NK (CD56+) cell counts and percentages (all *P* < 0.05).

**Fig. 7:**
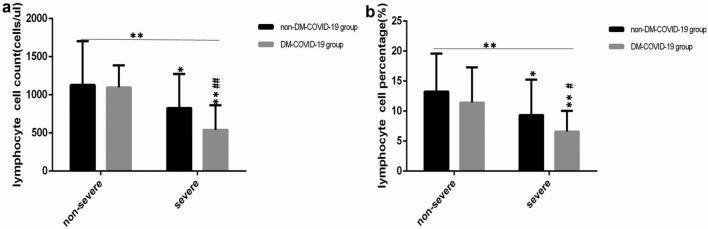
Comparison of lymphocyte levels and percentages among the four subgroups. DM, diabetes mellitus; non-DM, without diabetes mellitus; COVID-19, coronavirus disease 2019. **a** Lymphocyte count. **b** Lymphocyte percentage. Unpaired two-way ANOVA was used for interaction comparisons (**a**, **b**, *P* all > 0.05). Unpaired one-way ANOVA was used for intergroup comparisons (**a**, **b**, *P* all < 0.01). Unpaired *t*-tests was used for comparisons with the non-severe non-DM subgroup, *P < 0.05, **P < 0.01. Unpaired *t*-tests was used for comparisons with the severe non-DM subgroup, ^#^*P* < 0.05, ^##^*P* < 0.01

**Fig. 8:**
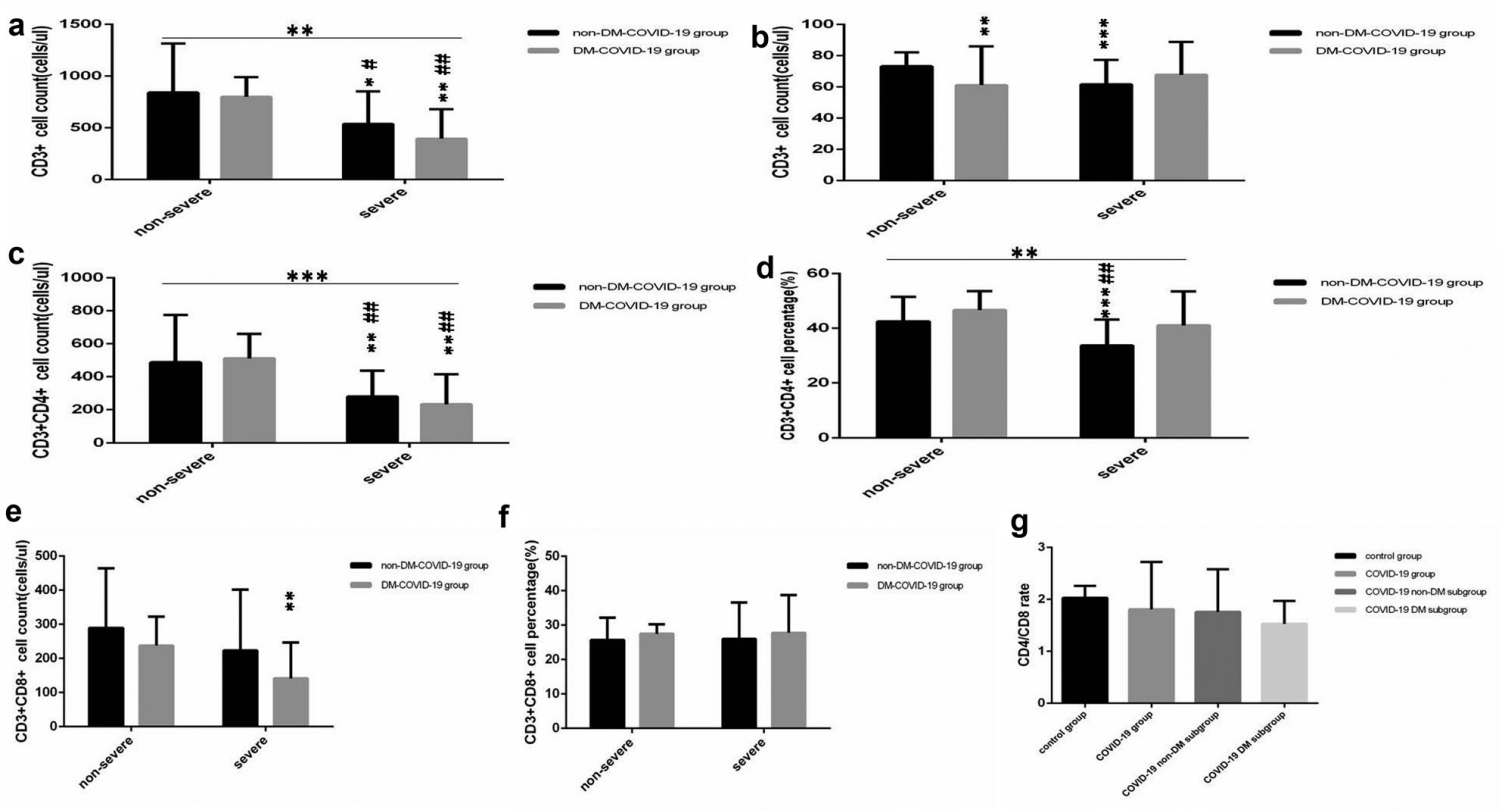
Comparison of T lymphocyte levels and percentages among the four subgroups. DM, diabetes mellitus; non-DM, without diabetes mellitus; COVID-19, coronavirus disease 2019. **a** CD3+ Cell count. **b** CD3+ Cell percentage. **c** CD3+ CD4+ cell count. **d** CD3+ CD4+ cell percentage. **e** CD3+ CD8 + cell count. **f** CD3+ CD8 + cell percentage. **g** Ratio of CD4 + /CD8 + cells. Unpaired two-way ANOVA was used for interaction comparisons (**b** *P* < 0.05; **a**, **c**–**g**, *P* all > 0.05). Unpaired one-way ANOVA was used for intergroup comparisons (**a**, **d**, *P* all < 0.01; **c** P < 0.001; **b**, **e**–**g**, *P* all > 0.05).Unpaired *t*-tests was used for comparisons with the non-severe non-DM subgroup, *P < 0.05, **P < 0.01, ***P < 0.001. Unpaired *t*-tests was used for comparisons with the severe non-DM subgroup, ^#^*P* < 0.05, ^##^*P* < 0.01

**Fig. 9:**
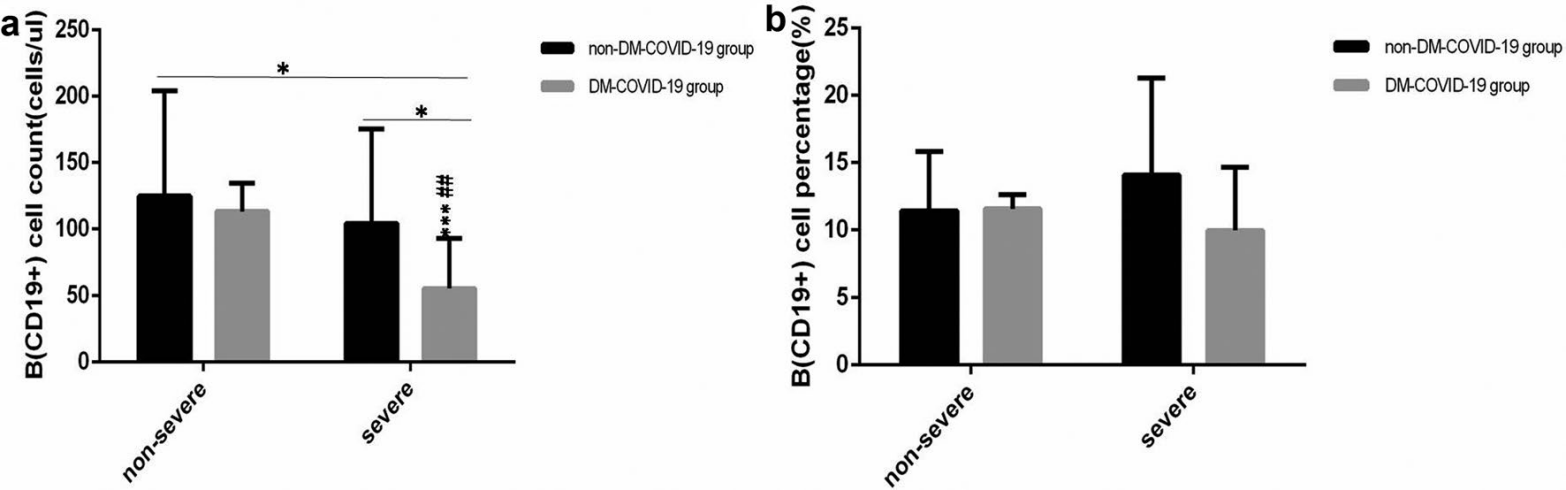
Comparison of B lymphocyte count and percentage among the four subgroups. DM, diabetes mellitus; non-DM, without diabetes mellitus; COVID-19, coronavirus disease 2019. **a** B lymphocyte count. **b** B lymphocyte percentage. Unpaired two-way ANOVA was used for interaction comparisons (**a**, **b** *P* all > 0.05). Unpaired one-way ANOVA was used for intergroup comparisons (**a** P < 0.05; **b** *P* > 0.05). Unpaired *t*-tests was used for comparisons with the non-severe non-DM subgroup or between two groups, *P < 0.05, ***P < 0.001. Unpaired *t*-tests was used for comparisons with the severe non-DM subgroup, ^##^*P* < 0.01

**Fig. 10:**
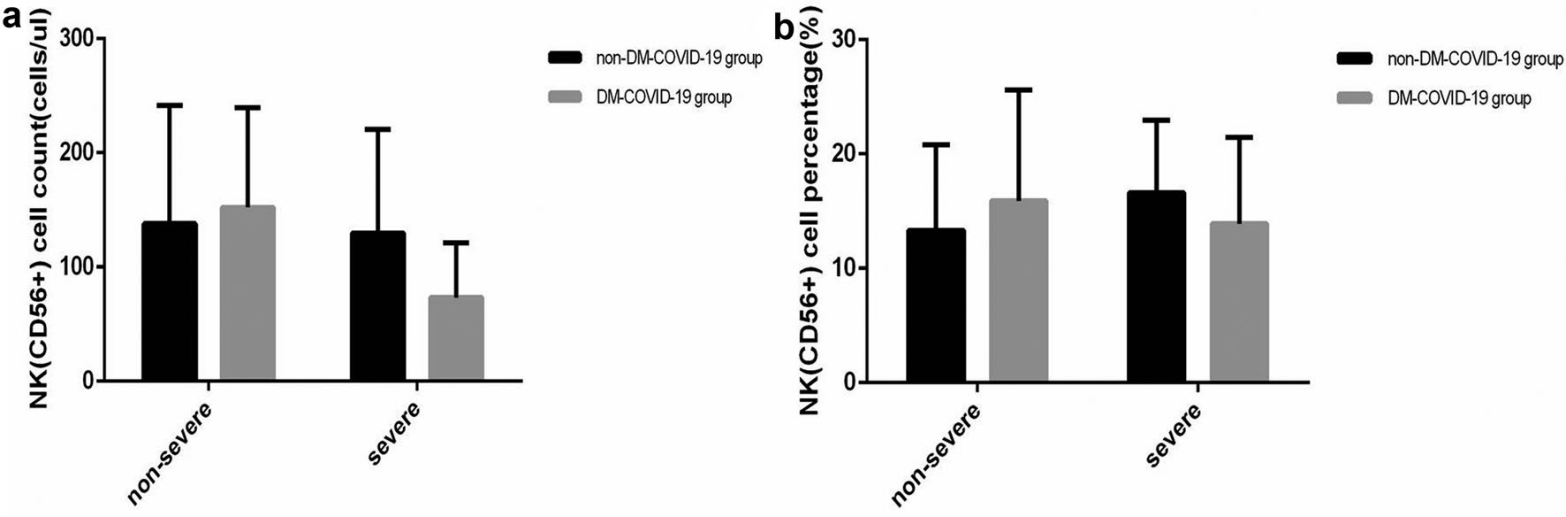
Comparison of NK lymphocyte count and percentage among the four subgroups. DM, diabetes mellitus; non-DM, without diabetes mellitus; COVID-19, coronavirus disease 2019. **a** NK lymphocyte count. **b** NK lymphocyte percentage. Unpaired two-way ANOVA was used for interaction comparisons (**a**, **b** *P* all > 0.05). Unpaired one-way ANOVA was used for intergroup comparisons (**a**, **b** *P* all > 0.05). Unpaired *t*-tests was used for comparisons with the non-severe non-DM subgroup or between two groups, *P* all > 0.05. Unpaired *t*-tests was used for comparisons with the severe non-DM subgroup, *P* all > 0.05

All lymphocyte and subset counts and lymphocyte percentages were similar in cases of non-severe COVID-19 regardless of whether or not the patients also had DM (Figs. 7a, b, 8a–f, 9a, b, 10a, b) (all *P* < 0.05).

Regardless of whether or not they had DM, patients with severe COVID-19 had lower lymphocyte counts and percentages (Fig. 7a, b), CD3+ Cell counts (Fig. 8a), CD3+ CD4+ cell counts (Fig. 8c), CD3+ CD8 + cell counts (Fig. 8e), B(CD19+) cell counts (Fig. 9a) than those with non-severe COVID-19 (all *P* < 0.05).

The lymphocyte counts and percentages (Fig. 7a, b), CD3+ cell counts (Fig. 8a), CD3+ CD4+ cell counts (Fig. 8c), CD3+ CD8+ cell counts (Fig. 8e), B(CD19+) cell counts (Fig. 9a) were the lowest in patients in the severe DM subgroup (all *P* < 0.05).

Regardless of whether or not patients had DM, and whether or not they had non-severe or severe COVID-19, the NK(CD56+) cell counts and lymphocytes (Fig. 10a, b) were similar (all *P* > 0.05).

### Disease progression and prognosis of COVID-19 patients

In the DM group, the prognosis was worse and the rate of severe disease was higher. The non-severe DM subgroup and the severe DM subgroup both had longer virus negative conversion times compared with the two non-DM subgroups, and the longest in-hospital time was found in the severe DM subgroup, these differences were all significant (Table 3).

**Table 3:** Comparison of the disease severity, the virus negative conversion time and the prognosis among the four subgroups (n = 95)

Variable	Non-DM group (*n* = 76)		DM group (*n* = 19)		*χ*^*2*^or *F* score	*P* score
	Non-severe subgroup (*n* = 57)	Severe subgroup (*n* = 19)	Non-severe subgroup (*n* = 8)	Severe subgroup (*n* = 11)		
virus negative conversion time	18.49 ± 10.02	20.53 ± 9.25	28.00 ± 12.84	27.73 ± 9.57	4.097	0.009
In-hospital time	14.25 ± 8.72	17.79 ± 12.33	19.38 ± 8.12	29.27 ± 16.59	6.704	< 0.0001
Severe(case, %)	19(25.00)		11(61.11)		-2.940	0.003
Prognosis					-3.394	0.001
Cured (case, %)	53(71.05)		5(26.32)			
Unhealed	21(26.32)		13(68.42)			
Death	2(2.63)		1(5.26)			

FPG, fasting plasma glucose; HbA1c, glycated hemoglobin A1c; DM, diabetes mellitus; non-DM, without diabetes mellitus

### The relationship of lymphocyte subsets and DM with disease progression and prognosis in COVID-19 patients

According to Spearman correlation analysis, age and DM were positively correlated, while lymphocyte counts and percentages, CD3+ Cell counts and percentages, CD3+ CD4+ cell counts and percentages, B(CD19+) cell counts and CD3+ CD8+ cell counts were all negatively correlated with disease severity (Table 4). The factors that were positively correlated with the viral negative conversion time included age and DM (Table 4). Furthermore, the disease severity, the coronavirus negative conversion time, DM and age were positively correlated, but the lymphocyte percentages was negatively correlated with prognosis (Table 4).

**Table 4:** Spearman correlation analysis of disease severity, virus negative conversion time, prognosis, baseline lymphocyte subset counts, age and DM(*n* = 95)

Variable	Disease severity (1 = common, 2 = severe, 3 = critically ill)		Virus negative conversion time(days)		Prognosis (1 = cure, 2 = unhealed, 3 = death)	
	r	p	r	p	R	p
DM (1 = without, 2 = with)	0.320	< 0.0001	0.337	0.001		
Age (year)	0.361	< 0.0001	0.264	0.010	0.263	0.010
LY (cells/μl)	–0.341	0.001				
LY% (%)	–0.371	< 0.0001			–0.209	0.042
CD3+ (cells/μl)	–0.379	< 0.0001				
CD3+ CD4+ (cells/μl)	–0.388	< 0.0001				
CD3+ CD8 + (cells/μl)	–0.351	< 0.0001				
CD+ 3% (%)	–0.302	0.003				
CD3+ CD4+ (%)	–0.219	0.033				
CD19 + (cells/μl)	–0.266	0.033				
Disease severity (1 = common, 2 = severe, 3 = critically ill)					0.331	0.001
Virus negative conversion time(days)					0.299	0.003

DM, diabetes mellitus; LY, lymphocytes

According to multiple step wise regression analysis for disease severity, the indicated factors included CD3+ CD4+ percentages, lymphocyte percentages, age and DM (Table 5). Moreover for the virus negative conversion time the indicated factors were B(CD19+) percentages and lymphocyte percentages (Table 5). Furthermore for the prognosis the indicated factors were the coronavirus negative conversion time, disease severity and age (Table 5).

**Table 5:** Multiple stepwise regression analysis of influencing factors of disease severity, coronavirus negative conversion time and prognosis (*n* = 95)

Independent variable		B	Std. Error	Beta	t	p
The disease severity (1 = common, 2 = severe, 3 = critically ill)	Constant	1.471	0.384	–	3.828	< 0.0001
	DM (1 = without, 2 = with)	0.537	0.185	0.266	2.897	0.005
	age	0.013	0.004	0.275	3.007	0.003
	CD3+ CD4 + %	– 0.023	0.007	– 0.287	– 3.236	0.002
	LY%	– 0.025	0.012	– 0.200	– 2.173	0.032
The coronavirus negative conversion time	Constant	18.421	3.173	–	5.805	< 0.0001
	CD19 + % (%)	0.394	0.184	0.255	2.134	0.037
	LY% (%)	– 0.413	0.171	– 0.290	– 2.422	0.019
The prognosis	Constant	0.33	0.161	–	2.067	0.042
	Coronavirus negative conversion time	0.022	0.006	– 0.354	4.005	< 0.0001
	Disease severity	0.220	0.063	0.316	3.481	0.001
	Age	0.007	0.003	0.204	2.141	0.035

DM, diabetes mellitus; LY, lymphocyte count

## Discussion

In this study, we found that the prevalence of DM in the COVID-19 group was 20% (19/95), and that the patients with severe COVID-19 had a higher the prevalence of DM than those with non-severe COVID-19. As a comorbidity in COVID-19 patients, [8, 9, 22] the 20%(19/95) prevalence of DM in this cohort was consistent with one study that reported a value of 20% (8/41) [8], which is higher than the 13% (13/99) reported in another study [9]. The 36.67% (11/30) prevalence of DM in patients with severe p COVID-19 compared with the 12.31%(8/65) in patients with non-severe COVID-19 was not consistent with the values reported I the literature; no significant difference in DM prevalence was found between patients with severe COVID-19 and those with non-severe COVID-19 [13]. The reason for this may be that, in this cohort, 8 DM cases were newly diagnosed after admission, and of these, 3 and 5 patients had non-severe and severe COVID-19. This discovery indicated that close monitoring of plasma glucose, assessment of glycated hemoglobin and a glucose tolerance test are necessary for COVID-19 patients to find those with DM in a timely manner.

After further analysis, we found that the rate of severity in patients with COVID-19 and DM was lower than that in those with COVID-19 and no DM. Moreover, DM was positively correlated with viral negative conversion time and disease severity, and DM was an essential influencing factor for disease severity. These findings were consistent with those reported in a 2003 study that found that in patients with acute respiratory distress syndrome (SARS) and DM, the mortality rate, and the rate of intensive care unit admission and mechanical ventilation were 3.0–3.3 times higher than the rates in those with no DM [23]. The rate of intensive care units admission in patients with H1N1 influenza and DM was 4.29 times higher than that in patients without DM, [24] and in a 2014 study, DM was also a high-risk factor for severe Middle East Respiratory Syndrome coronavirus infection [24].

We also found that lymphocytes in general as well as lymphocyte subset were significantly reduced in COVID-19 patients, especially those with DM. Moreover, the rate of severity was the highest in those with DM, the prognosis was worse, and the lymphocyte counts and lymphocyte subset counts, especially CD4+ T cells, CD8+ T cells and B cells were the lowest in those with severe COVID-19 and DM, these findings were consistent with the literature [13, 25, 26]. However, in the literature, no hierarchical analysis was performed between COVID-19 patients with and without DM, [13, 25] and no hierarchical analysis was performed between non-severe and severe COVID-19 patients with or without DM [26].

After further analysis, we found that lymphocytes and lymphocyte subsets were negatively correlated with disease severity, and that lymphocytes were negatively correlated with prognosis. The factors associated with disease severity were lymphocyte percentages, CD4+ cell percentages, age and DM, and additionally, disease severity, age and the virus negative conversion time were associated with prognosis.

We also found that in severe cases of COVID-19 and DM, the B (CD19+) cell counts were the lowest and the virus negative conversion time was the longest. The factors associated with virus negative conversion time were B(CD19+) cell percentages and lymphocyte percentages, which was not consistent with the literature [13] in which no difference in B(CD19+) cell percentages or counts were found between non-severe and severe COVID-19 patients.

In this study, we also found that patients with severe COVID-19 without DM were significantly older than patients with non-severe COVID-19 without DM, which was consistent with the poor prognosis found in elderly COVID-19 patients [8, 9]. One study found that the CD4 T cell subsets was markedly different between old and young mice, three cell subsets were terminally exhausted, and cytotoxic as well as activated regulatory T cells (Tregs) rarely appeared in young mice, but they gradually accumulated with age. The extreme anti-inflammatory and pro-inflammatory phenotypes of cytotoxic CD4+ T cells and Tregs were most unexpected [27]. It was found that the relative frequency and total number of B cells will decrease with age. Plasma blasts, memory cells and transitional B cells are decreased in patients older than 70 years [28]. Lymphocytes and their subsets [including NK cells (CD56+), B cells (CD19+) and T cells (CD3+)] are mainly responsible for regulating host immunity. T cells play an important role in promoting or maintaining inflammation by producing inflammatory cytokines [24, 29–32]. A subtype of CD4+ effector T cells consists of activated Th1 cells, which trigger phagocyte-dependent inflammation and cell-mediated immunity through the production of Interferon-γ (IFN-γ), interleukin 2 (IL-2) and tumor necrosis factor β (TNF-β) [24]. In contrast, another subtype of CD4+ effector T cells, activated Th2 cells, modulat the antibody response by producing IL-13, IL-10, IL-9, IL-6, IL-5 and IL-4 [29]. Viral infection play a major role in disease progression by inducing an indirect host immune response and direct cytopathic effects [13, 30]. A rapid and well-coordinated innate host immune response is the first line of defense against viral infections, but a dysregulated immune response could lead to excessive inflammation and even death [13].

Patients with non-severe COVID-19 without DM were younger than those with severe COVID-19 without DM, and age was positively correlated with prognosis, viral negative conversion time and disease severity. Moreover, age was an essential determining factor of prognosis and disease severity, which was consistent with the finding that elderly patients had a poor prognosis [8, 9]. A similar age difference was not found between patients with non-severe COVID-19 and DM and those with severe COVID-19 and DM, which was inconsistent with previous reports [8, 9]. Type 2 diabetes mellitus (T2DM) is a systemic chronic low-grade inflammatory disease. The function of specific T lymphocyte subsets (including regulatory T (Treg) cells) changes, leads to the following hypothesis: partial inflammation exacerbates T2D autoimmunity [33]. T cells play an important role in promoting or maintaining insulin resistance and inflammation by inducing the production of pro-inflammatory cytokines in metabolic organs (such as the pancreas, muscle, adipose tissue and liver) [32, 34–36]. In adipose tissue, the major inflammatory cells are macrophages [32, 34–36]. Proinflammatory M1 macrophages releases proinflammatory cytokines, such as IL-6, TNF-a and IL-1, which contribute to local and systemic inflammation [36]. On the contrary, anti-inflammatory M2 macrophages secrete IL-10, which inhibits the activity of most pro-inflammatory cell types including M1 macrophages [36]. By interacting with the p38/MAPK pathway, IL-10 suppresses TNF-α [36]. Th1 cells that produce TNF-α, IL-2 and IFN-γ promote M1 polarization and enhance the pro-inflammatory functions of M1 macrophages. In contrast, Th2 cells skew the differentiation of macrophages towards M2 by producing the anti-inflammatory cytokines IL-4, and IL-13 [32, 34–36]. Therefore, Th1 and Th2 responses, which are closely related to M1/M2 polarization, may also play a critical role in T2DM [32, 34–36].

Previous research found a higher expression of proinflammatory cytokines and chemokines in COVID-19 patients, especially in severe cases. the exhaustions of CD4+ and CD8+ T cells, and the decrease in regulatory T cells, might result in aggravated inflammatory responses, and the resultant cytokine storm and may exacerbate damaged tissue. A lower number of lymphocytes suggests a role for dysregulated immune responses in COVID-19 pathogenesis [13, 14]. Our research has suggested that the coexistence of viral infection and DM results in more dysregulated host immune responses, which worsens the already aggravated inflammatory process. Patients are thus, more susceptible to bacterial infections, more severe organ damage and a worse prognosis. The coexistence of viral infection and DM can also reduce or delay antibody production by decreasing the B cells (CD19+) count and percentage, thereby delaying the removal of the virus and leading to a worse prognosis.

Our study had several limitations. First, it was a retrospective, single-center and small-sample study. Second, CD4 + effector T cell subtypes (e.g., pro-inflammatory Th1 and Th17 cells, and anti-inflammatory Th2 and Foxp3 + regulatory T cells (Tregs)), and the two populations of macrophages (pro-inflammatory M1 and anti-inflammatory M2) were not further identified. Third, the production time and dynamic characteristics of SARS-CoV-2 specific IgM and IgG antibodies could not be obtained. Despite these limitations, our study demonstrated several novel details in that the coexistence of viral infection and DM results in a more dysregulated host immune responses and thus worsens the already aggravated inflammatory process. Those patients are, more susceptible to bacterial infections, more severe organ damage and a worse prognosis. The coexistence of a viral infection and DM can reduce or delay antibody production, thereby delaying removal of virus and leading to a worse prognosis.

## Conclusions

The patients with severe COVID-19 and DM had the lowest numbers of lymphocytes, especially T and B lymphocytes. Lymphocyte subsets were decreased and DM maybe have led to a poor prognosis by affecting the disease severity and prolonging the viral negative conversion time. Combination immunomodulatory therapy based on comprehensive treatment might improve disease progression and prognosis in COVID-19 patients, especially those with severe COVID-19 and DM.

## Data Availability

All data, models, or code generated or used during the study are available from the corresponding author by request: Dafeng Liu, E-mail: liudf312@126.com.
